# Large-scale reconstruction of 3D structures of human chromosomes from chromosomal contact data

**DOI:** 10.1093/nar/gkt1411

**Published:** 2014-01-24

**Authors:** Tuan Trieu, Jianlin Cheng

**Affiliations:** ^1^Computer Science Department, University of Missouri-Columbia, MO 65211, USA, ^2^Informatics Institute, University of Missouri-Columbia, MO 65211, USA and ^3^C. Bond Life Science Center, University of Missouri-Columbia, MO 65211, USA

## Abstract

Chromosomes are not positioned randomly within a nucleus, but instead, they adopt preferred spatial conformations to facilitate necessary long-range gene–gene interactions and regulations. Thus, obtaining the 3D shape of chromosomes of a genome is critical for understanding how the genome folds, functions and how its genes interact and are regulated. Here, we describe a method to reconstruct preferred 3D structures of individual chromosomes of the human genome from chromosomal contact data generated by the Hi-C chromosome conformation capturing technique. A novel parameterized objective function was designed for modeling chromosome structures, which was optimized by a gradient descent method to generate chromosomal structural models that could satisfy as many intra-chromosomal contacts as possible. We applied the objective function and the corresponding optimization method to two Hi-C chromosomal data sets of both a healthy and a cancerous human B-cell to construct 3D models of individual chromosomes at resolutions of 1 MB and 200 KB, respectively. The parameters used with the method were calibrated according to an independent *fluorescence in situ hybridization* experimental data. The structural models generated by our method could satisfy a high percentage of contacts (pairs of loci in interaction) and non-contacts (pairs of loci not in interaction) and were compatible with the known two-compartment organization of human chromatin structures. Furthermore, structural models generated at different resolutions and from randomly permuted data sets were consistent.

## INTRODUCTION

The 3D organization of a genome was found to play an important role in gene–gene interaction, gene regulation and genome methylation (1–4). For instance, it was shown that genes at long sequential genomic distances could functionally interact through physical spatial contacts (5), often leading to long-range gene regulation and collaboration. Understanding 3D chromosomal structures is essential for decoding and interpreting functions of a genome as whole and its functional and regulatory elements (e.g. genes and transcription factor binding sites). However, owing to lack of experimental techniques of directly determining the 3D shape of a genome consisting of billions of nucleotides, little is known about the 3D organization of a genome and its largest discrete components—chromosomes.

Recently, chromosome conformation capture (3C)-based techniques have emerged as powerful tools for capturing physical interactions (e.g. spatial contacts) between pairs of chromosomal regions (e.g. loci) (6) on the same or two different chromosomes. Particularly, an advanced 3C technique—Hi-C—has been developed to determine both intra- and inter-chromosomal contacts at a genome scale rather uniformly and unbiasedly (7), which provides crucial information necessary for studying and reconstructing the 3D shape of a chromosome or genome for the first time.

Therefore, some computational methods have been developed to reconstruct the 3D shapes of chromosomes and genomes from chromosomal contact data. In (8), interaction (contact) frequencies between loci were converted into Euclidian distances, which were then used as distance constraints between loci being solved by a constrained optimization method to obtain the coordinates for loci in the 3D space. Similarly in (9), the converted distances between loci were used by a Markov chain Monte Carlo (MCMC) sampling technique to reconstruct structures that satisfy as many distance constraints between loci as possible.

Despite being highly valuable, the pioneering methods based on converted distances may still have some limitations. First, the distances converted from chromosomal interaction frequencies may not be accurate due to various reasons, such as biases in techniques to capture interaction frequencies and non-uniform relationships between distances and interaction frequencies. Second, some 3D models reconstructed by the distance-based methods still do not exhibit some important known features of chromatin organization.

To overcome these problems, here, we present a novel method to reconstruct the 3D structure of a chromosome directly from chromosomal contacts extracted from the Hi-C data in (7) without converting chromosomal interaction frequencies into distances. The method aims to build most likely (or preferred) 3D chromosome structures that can satisfy the chromosomal contacts with higher probability directly while obeying necessary physical constraints such as contact distance thresholds and maximum/minimum distances between two chromosomal regions.

## MATERIALS AND METHODS

We used the Hi-C data of the normal B-cell GM06990 (7) and the malignant B-cell of an acute lymphoblastic leukemia patient (10). The data were pre-processed as follows before they were used to build 3D models for the 23 pairs of human chromosomes.

### Data normalization

Because there are several sources of biases in Hi-C experiments, such as cutting frequencies of restriction enzymes, GC content and sequence uniqueness (11), data normalization is necessary. We used a simple data normalization protocol (7,8,12) to pre-process the Hi-C data. Given an initial n × n interaction frequency (IF) matrix C representing contact numbers between n units (e.g. regions of equal size) of a chromosome that were generated from a raw Hi-C data set, an element 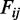 denoting IF between regions 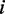 and 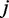 of a chromosome in a normalized matrix 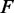 is calculated according to the formula below.
(1)
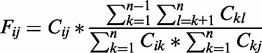



The first term, 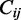 in the initial IF matrix, is the observed number of Hi-C reads linking regions 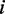 and 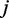. The inverse of the second term in the formula is the expected IF between two regions 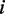 and 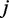, which is used to normalize 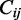. The expected IF is the product of the number of interactions (contacts) of each region divided by the total number of interactions (contacts). The normalized IF (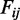) is obtained by dividing the observed IF (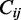) by the expected IF. This normalized IF could be interpreted as the likelihood ratio (odds) of regions 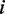 and 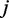 in contact, which is the number of observed reads divided by the number of expected reads between the two regions. Thus, the normalized IF represents how likely regions *i* and *j* are in contact with respect to the expected chance. Contacts with low likelihood ratio have a low normalized IF and contacts with high likelihood ratio have a high normalized IF.

### Removal of contacts with low contact likelihood ratio

The normalized contact matrices of individual chromosomes calculated from the data in (7) at the resolution of 1 MB (i.e. unit size is 1 M base pairs) are 95–100% full of non-zero values, which means if chromosomes are divided into regions of 1 MB, almost all the regions in a chromosome have at least one contact with the other regions. However, because the range between the minimum and maximum interaction frequencies is large, contacts with low interaction frequencies could be noisy or less likely transient interactions due to the ‘dynamic’ property of chromosome structures, and thus could be practically removed before constructing preferred 3D structures. In fact, our method always tries to satisfy contacts with high probabilities (likelihood ratio) first, so considering contacts with low probabilities or not does not influence structural models significantly. We determined an IF cutoff value to remove potential noisy or unlikely contacts based on a known feature of chromosome organization that larger chromosomes lie near the nuclear periphery and small chromosomes (chromosomes 16, 17, 19, 20, 21 and 22) co-localize in the center of the nucleus (7). According to this feature, there should be no or few inter-chromosomal contacts between chromosomes lying near the nucleus periphery and those lying in the center of nucleus. However, in the raw Hi-C data in (7), >95% inter-chromosomal region pairs are in contact before filtering out noise (see Supplementary Table S1 for the percentage of region pairs in contact for all chromosome pairs), suggesting chromosomes are entangled together, which conflicts with the fact that each chromosome largely occupies its own spatial territory (7). Thus, we tested different IF cutoff thresholds and chose a value that seemed to satisfy the feature the best. The selected IF cutoff value on normalized interaction frequencies is 0.66. The percentage of region pairs in contact between all chromosome pairs and within each chromosome after applying the threshold to remove noise is shown in Supplementary Table S2. Interestingly, this matrix shows that chromosome 18, which is a small chromosome and is supposed to lie near the periphery, has the higher percentage of contacts with chromosome 21 than chromosomes 16 and 17. Using this cutoff threshold, we obtained intra-chromosomal contacts for each chromosome. The percentages of intra-chromosomal contacts of all the chromosomes are listed on the diagonal of the matrix in Supplementary Table S2. We observed that the percentages of intra-chromosomal contacts of larger chromosomes (chromosomes 1–7) are relatively low, suggesting that larger chromosomes spread wider than smaller chromosomes do.

### Representation and construction of chromosome structures

To represent and build the 3D structure of a chromosome, we divide its sequence into sub-regions of equal size. The size of the region (e.g. 1 Mb) determines the granularity of the structure. Larger the size, lower is the resolution. The position of a region is represented by its midpoint with three coordinates. The chromosome is then modeled as a continuous piece-wise linear curve in the 3D space connecting the midpoints of all the regions together. Because there were no sequence reads mapped to the centromeres of chromosomes in the Hi-C data sets, the centromere regions were not included into the model.

**Figure 1.**
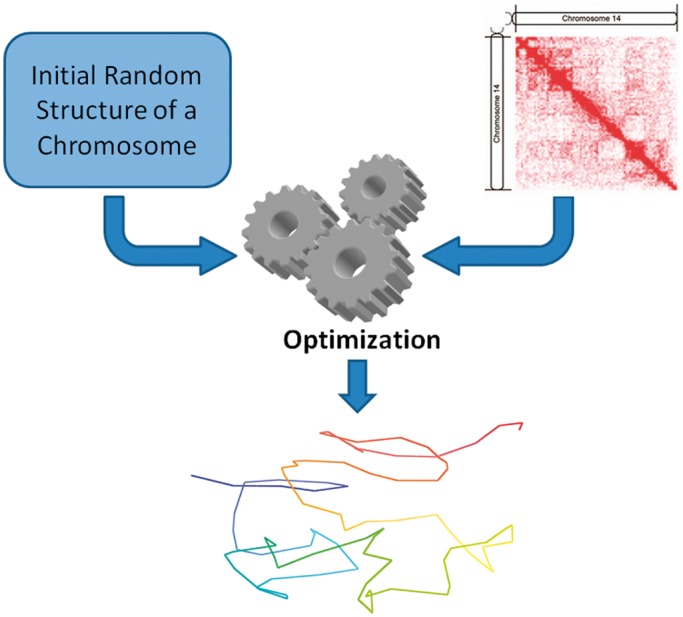
The flowchart of constructing a chromosome model from chromosomal contact data. Given an initial random structure of a chromosome and the chromosomal contacts observed in the Hi-C data, a computational optimization method is used to tweak the structure to maximize the observed contacts in the structure while reducing false contacts according to an objective function.

In a 3D model of a chromosome, two regions are considered in contact if the Euclidean distance between their midpoints is less than a distance threshold 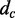, and pairs of these regions are called contacts. Otherwise, the two regions are not considered in contact, and pairs of these regions are called non-contacts. Our goal is then to place the regions of a chromosome in the 3D space to satisfy contacts and non-contacts observed in the region–region contact map generated from Hi-C data sets of the chromosome as much as possible, which is a constrained spatial optimization problem (see Figure 1 for an illustration of the optimization problem).

The goal of optimization is to make the distances between region pairs in contact less than 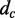 (contact distance threshold) and the distances between non-contact regions at least 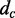. We define an objective function (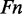) of the distances of all pairs of regions to achieve the goal as follows.
(2)
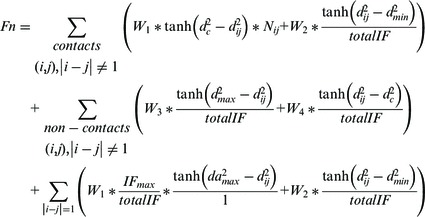



Here 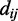 is the distance between midpoints of 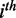 and 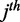 regions; 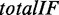 is the total IF of the chromosome; and 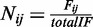 is the normalized IF between regions 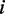 and 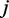 divided by total number of IF. Here 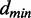 and 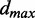 are the minimum and maximum distances between two regions, respectively, 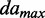 is the maximum distance between two adjacent regions and 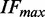 is the maximum IF among all pairs. 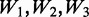 and 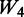 are chromosome-dependent adjustable parameters for maximizing the number of satisfied contacts and non-contacts. 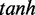 is the hyperbolic tangent function. The coordinates of points in the initial structure of a chromosome were randomly initialized in the range (−0.5 to 0.5). We then maximized the objective function by using steepest gradient ascent with the backtracking line search algorithm (13) to adjust the (x, y, z) coordinates of chromosomal regions. (A video that demonstrates how the optimization process works is available at http://www.youtube.com/watch?v=C03R7A9kYc8).

The first term in formula (2) enforces the distance constraints on contacts and also their minimum distance, i.e. the objective function increases as the distance between contacted regions gets smaller than 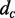 or larger than 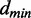. The second term enforces the constraints on non-contacts and their maximum distance, i.e. the objective function increases as the distance between non-contacted regions gets larger than 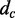 or becomes smaller than 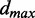. The last term exerts forces on the distance of two adjacent points to make them less than 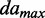 and greater than 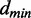. We use 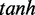 function to smooth the difference between a distance and a distance threshold. One benefit of using 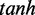 function is that its derivative can easily be calculated, which is desirable for the gradient ascent-based optimization. 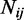 in the first term weights the influence of a contact proportionally according to its normalized IF, i.e. a contact with higher IF contributes more to the value of the objective function. Parameters 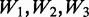 and 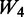 are weights assigned to the components in the three terms.

### Parameter estimation

To evaluate generated models, we defined a contact score as the percentage of satisfied contacts over all contacts and a non-contact score as the percentage of satisfied non-contacts over all non-contacts in the Hi-C data sets. We expect that in good models, both scores are high. It might happen that one score is high while the other is low. But if the two scores are balanced, both could be high. Parameters 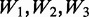 and 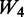 in formula (2) are adjusted to balance and maximize both scores. Different chromosomes may have different values for 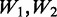, 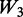 and 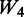. To determine these values for chromosomes, we tried different values around the average IF of the chromosome. Generally, increasing 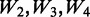 will increase non-contact score while decreasing contact score and increasing 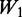 will increase contact score while decreasing non-contact score. Supplementary Table S3 lists the values of these four parameters used for all chromosomes at 1 MB resolution.

The parameters 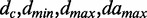 in formula (2) are estimated from the fluorescent *in situ* hybridization (FISH) chromosomal distance data in (14). The FISH data contain spatial distances between some regions of the human chromosomes at various genomic distances. We used the spatial distance of regions at genomic distance of ∼1 MB to estimate 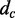, 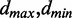 and 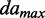. The minimum and maximum distances measured for pairs of regions ∼1 MB away from each other were used to approximate 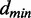 and 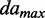. We set 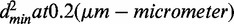 and 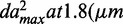) for all chromosomes.

Theoretically, 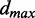 (i.e. the maximum distance between any two intra-chromosomal regions) could be equal to the diameter of the nucleus. However, because a chromosome folds into its own territory, 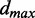 should be well below the diameter of the nucleus. Because the maximum distance measured in the FISH data is ∼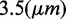, we set 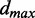 to 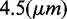 for all chromosomes allowing some room of variation. During the modeling process, the value of 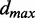 was important only for large chromosomes (chromosomes 1–7), where the percentage of intra-chromosomal contacts was relatively low, while the models constructed for other smaller chromosomes with the larger percentage of contacts were not sensitive to 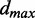 values >4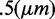.

**Figure 2.**
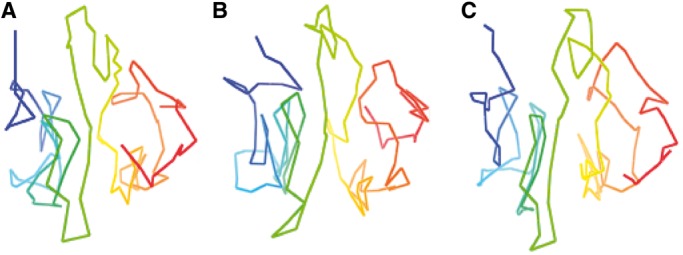
The models for chromosome 11 constructed at different contact threshold 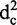 of 6 µm (**A**), 7 µm (**B**) and 8 µm (**C**). The blue part is the beginning of the chromosome and the red part is the end of the chromosome.

We estimate 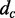 based on the average distance of pairs of regions measured in the FISH data. At resolution of 1 MB, the percentage of intra-chromosomal contacts in chromosome 11 is ∼77%, which means that most of region pairs are in contact. Thus, we tried to make the average distance of all pairs in our generated models for chromosome 11 approximately equal to the approximate average distance of all pairs of regions measured in the FISH data. We found that setting 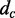 a little larger than the approximate average distance could satisfy this requirement. Therefore, we set 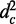 to 7.0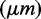 for chromosome 11 and all other chromosomes. We also observed that our method is not sensitive to the fluctuation of 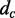 value. For instance, changing the range of 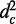 within 1 U of the current value did not affect generated structures substantially. Figure 2 illustrates the structures of chromosome 11 constructed at three difference contact thresholds.

## RESULTS

We validated chromosomal structural models using both various scoring functions and known physical features of human chromosomes such as the two-compartment feature (7). The evaluation protocol and results are described later in the text.

### Selection of a representative model from an ensemble of models and the consistency checking of ensembles

For each chromosome, we generated an ensemble of 300 models. We used a clustering approach widely used in protein model selection (15) to select a representative model for each ensemble. We modified the protein structure comparison program TM-Score (16) to calculate the structural similarity between every pair of chromosomal models. Because the standard TM-Score was originally designed to score protein structures with distance thresholds used for calculating the percentage of aligned residue–residue pairs that are too large to distinguish the difference between chromosomal models, we lowered its four thresholds from 4.0, 2.0, 1.0 and 0.5 to 2.0, 1.5, 1.0 and 0.5, respectively, to compute the average percentage of pairs of regions whose distance is below these thresholds after two chromosomal models are superimposed. This average percentage called the global distance test—high accuracy (GDT-HA) score according to the nomenclature of protein structure comparison (14) measures what percentage of regions of two chromosome models are superimposable, i.e. whose spatial positions are similar to each other. We defined the distance between two chromosomal models as the inverse of the GDT-HA score between them. Using the K-medoids algorithm (17), we identified the centroid model of the ensemble as its representative model.

**Figure 3.**
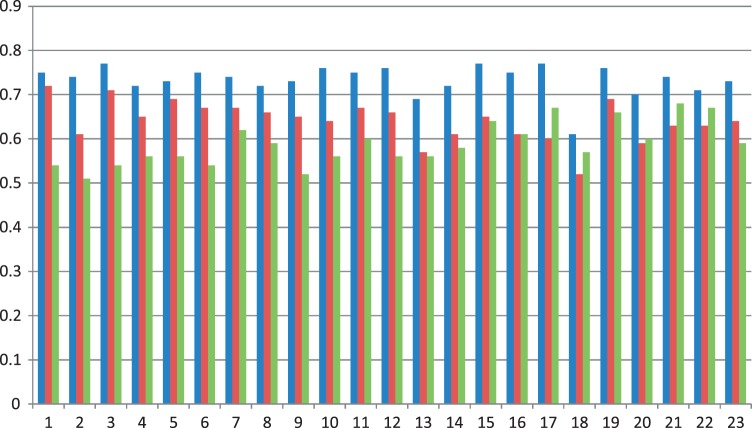
The similarity scores measured as average GDT-HA scores. Y-axis denotes the similarity scores and X-axis the indices of chromosomes. ‘Blue bars’ represent the average GDT-HA scores of models within the same model ensemble constructed from the whole normalized data sets of the normal B-cell for each chromosome, ‘red bars’ the average GDT-HA scores between models constructed from sampled data sets with those constructed from the whole data sets and ‘green bars’ the average GDT-HA scores between models of the leukemia B-cell and those of the normal B-cell.

To gauge how similar models constructed in ensembles for each chromosome are, we calculated average GDT-HA score of all pairs of models in an ensemble to see how different the structures are. This score is considered as the pairwise similarity of models within an ensemble. The blue bars in Figure 3 report the average similarity scores of the models for all 23 chromosomes, respectively. The average GDT-HA scores of all the chromosomes except chromosomes 13 and 18 are >0.7, suggesting that the models in an ensemble are similar despite different initializations and optimization trajectories.

**Figure 4.**
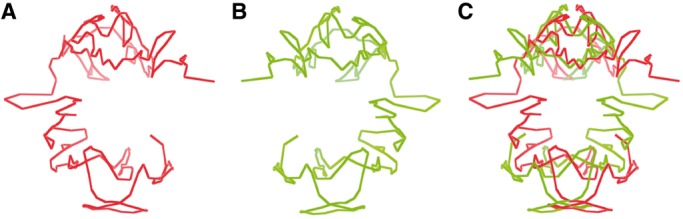
(**A**) A structural model of chromosome 1; (**B**) a mirror structure of structure (A); and (**C**) The superimposition of the strucutre in (A) and its mirror (B).

Closely comparing structural models in an ensemble, we observed that most difference between models in an ensemble comes from some parts in models that are the mirror of each other. Figure 4 shows two models of chromosome 1 that have some parts mirroring each other, which cannot be distinguished by distance/contact information alone. This is reasonable from the geometric point of view, as they both satisfy the geometric constraints imposed.

### Validation of models by various scoring measures

We evaluated the representative models of all the chromosomes by the percentage of contacts/non-contacts satisfied at first. The results show that the representative models of all the chromosomes satisfied at least 87% of contacts and 83% of non-contacts. Columns 2 and 3 of Table 1 show the scores for all chromosomes.

**Table 1.** The summary of the evaluation of 23 chromosome models

Chromosome	Contact score (%)	Non-contact score (%)	Average squared distance (unsatisfied contact pairs) 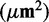	Average IF (unsatisfied contact pairs)	Average squared distance (unsatisfied non-contact pairs) 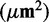	Average IF	Percentage of satisfied IF (%)
1	87	84	10.46	0.78	5.07	2.63	96.3
2	87	83	10.73	0.78	5.13	2.52	96.2
3	87	83	9.56	0.82	5.30	2.47	95.7
4	90	87	9.99	0.82	4.55	2.89	97.2
5	88	86	10.16	0.81	4.75	2.84	96.6
6	89	84	10.21	0.83	4.87	2.77	96.6
7	85	84	10.98	0.84	4.96	2.91	95.8
8	90	86	10.46	0.83	4.74	2.98	97.2
9	89	89	9.68	0.85	4.87	3.49	97.3
10	88	87	10.32	0.84	4.89	2.94	96.6
11	87	88	9.66	0.85	5.02	2.74	96.2
12	87	89	10.73	0.85	4.91	2.95	96.4
13	90	91	9.28	1.01	4.45	3.69	97.4
14	91	93	9.41	0.91	3.13	3.59	97.7
15	92	92	8.8	0.87	3.94	3.57	98.2
16	91	93	9.02	1.01	4.11	3.95	97.7
17	91	94	9.24	0.91	4.66	3.56	97.7
18	91	98	9.18	1.15	2.80	3.95	97.4
19	95	98	8.55	0.92	4.45	5.38	99.2
20	95	92	8.49	0.92	3.77	4.14	98.9
21	95	97	9.01	0.93	2.66	7.04	99.3
22	98	95	8.47	0.91	2.97	6.01	99.7
23	88	89	10.7	0.86	4.99	3.04	96.7

Columns 2–8 list the percentage of satisfied contacts, the percentage of satisfied non-contacts, the average squared distance of unsatisfied contact pairs, the average IF of unsatisfied contact pairs, the average squared distance of unsatisfied non-contact pairs, the average IF of each chromosome and the percentage of satisfied interaction frequencies for 23 chromosome models. The squared contact distance threshold is 7. The results show that the average IF of unsatisfied contacts (column 5) is much smaller than the average IF in a chromosome (column 7).

We also observed that the contacts that were not satisfied by the models were mostly those with lowest interaction frequencies. Therefore, we investigated how serious the violations for unsatisfied contacts and non-contacts are. We calculated the average IF and the average spatial distance of contact pairs that were not satisfied (Table 1). Their average IF was small and the average squared spatial distance was close to 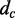
^2^ (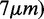, which indicates that the least likely contacts were not satisfied and the distance violations were often not serious. Similarly, we measured the average squared spatial distance of unsatisfied non-contacts, which is smaller than, but close to 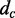
^2^, suggesting that the violation of unsatisfied non-contact pairs was not serious either. Table 1 reports the average squared distance of unsatisfied contacts, average IF of unsatisfied contacts, the average squared distance of unsatisfied non-contacts and average IF of all contact pairs of all the chromosomes (for comparison purpose). Furthermore, we calculated the percentage of total IF that is satisfied, which is the sum of IFs of satisfied contacts divided by the total IF (the last column in Table 1). The percentage of satisfied IF for all the chromosomes was always >95%, indicating the interaction frequencies in the Hi-C data were well preserved in the models.

### Validation of chromosomal models by the two-compartment feature

**Figure 5.**
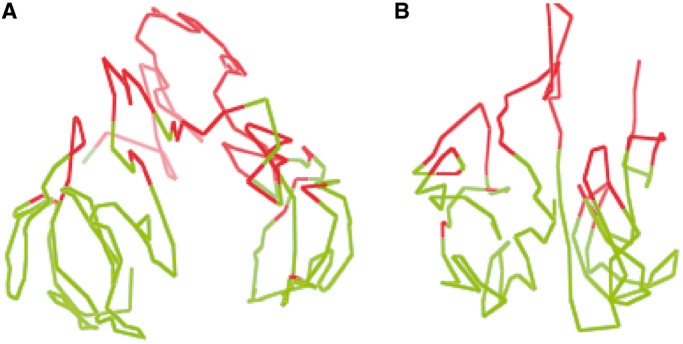
Two compartments in chromosome 1 (**A**) and chromosome 11 (**B**) were identified by the principal component analysis on contact maps and colored in different colors in the 3D models.

It is known that the human chromosomes are partitioned into two separated compartments (euchromatin and heterochromatin), where there are more intra-compartment contacts than inter-compartment ones (7). To test whether this feature exists in our chromosomal models, we first performed the principal component analysis on the chromosome contact maps to identify chromosomal regions partitioned into two compartments, and then highlighted the regions assigned to two compartments in the models using two different colors (euchromatin in red and heterochromatin in green). We found that the regions in the same compartment were also spatially clustered together in the models, and the two compartments largely occupied separate spaces, although they might not be completely separable. Figure 5 illustrates the two compartments assigned according to contact maps in the models of chromosomes 1 and 11. Regions in the same compartments tend to be close together, although they may have larger genomic distances (i.e. number of base pairs separating them). This confirms that genomic regions or genes far away on the genome sequence may spatially interact in the 3D space (5). Supplementary Figure S2 visualizes the two compartments of all chromosomal models at 1 MB resolution.

**Figure 6.**
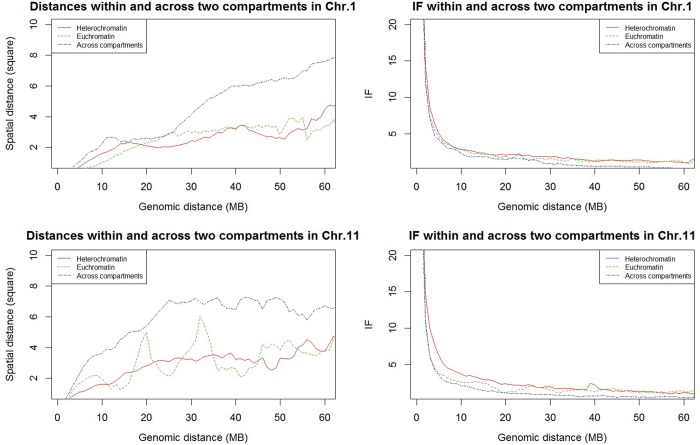
Average of spatial distances and IFs of region pairs within and across (between) compartments in chromosomes 1 and 11. Red: region pairs in heterochromatin compartment; green: region pairs in euchromatin compartment; and blue: region pairs across/between two compartments.

Figure 6 shows average spatial distances and IFs of pairs of regions at different genomic distances within the same compartment and across/between compartments. For chromosome 1, interaction frequencies in the heterochromatin compartment are only slightly higher than those in the euchromatin compartment, resulting in shorter spatial distance in heterochromatin in the structure for only some region pairs. For chromosome 11, interaction frequencies in the heterochromatin compartment are substantially higher than those in the euchromatin compartment for regions <10 MB genomic distance, resulting in a much shorter spatial distance between those regions in the structure.

### Method robustness testing

**Figure 7.**
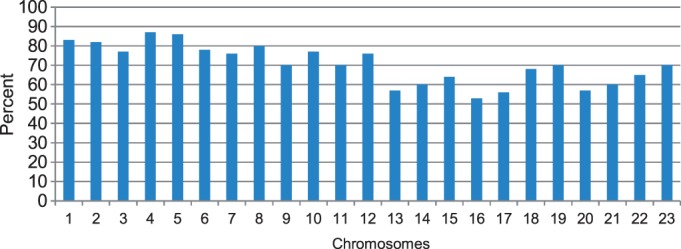
The percentage of recovered contacts in all chromosomes.

To assess how well the reconstruction method can recover the missing contacts not present in the input Hi-C data, we randomly sampled 70% contacts while withholding the other 30% contacts as non-contacts to make a new data set for each chromosome. We shall call these data sets as ‘sampled datasets’ to distinguish them from the whole data sets containing all observed contacts. We constructed 3D models for chromosomes using the sampled data sets and counted how many (or percentage of) contacts among 30% withheld contacts were satisfied, which estimates the percentage of unseen contacts that could be recovered in the constructed model. The percentage of contacts that are recovered is in the range of 53–86%, suggesting that our reconstruction protocol can predict unseen contacts reasonably well. We also calculated the average GDT-HA score (i.e. similarity score) between the models of each chromosome constructed from the sampled data set with that of the models generated from the whole data set. The average GDT-HA scores were relatively high (see red bars in Figure 3) and close to the similarity scores of the models in the same ensemble constructed from the whole data set for each chromosome, indicating our method is robust against the missing contacts. The percentages of recovered contacts are shown in Figure 7. It is interesting that higher percentages of contacts in the larger chromosomes tended to be recovered than in the smaller chromosomes.

**Figure 8.**
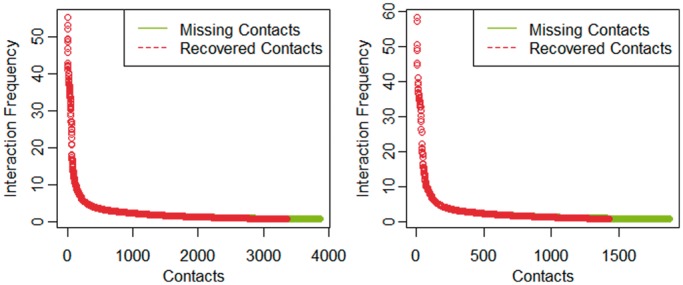
The superimposition of the plots of IFs of all missing contacts and the plots of IFs of recovered contacts for chromosome 1 (left) and chromosome 11 (right). Y-axis denotes interaction frequencies and X-axis the indices of contacts. The green tails visualized the contacts that were not recovered and had lower IFs.

We further checked the recovered contacts in details and observed that recovered contacts were the ones with higher IF and the ones that were not recovered had lower IF. This suggests that stronger interactions were more likely recovered. We sorted all IFs of the 30% withheld contacts and all IFs of truly recovered contacts in descending order for chromosomes 1 and 11, which are plotted and superimposed in Figure 8. Supplementary Figure S1 shows the plots for all chromosomes. It is shown that the missing contacts (green) with high IFs overlapped well with the recovered contacts (red).

### Comparison of models constructed at 200 KB with those at 1 MB

**Figure 9.**
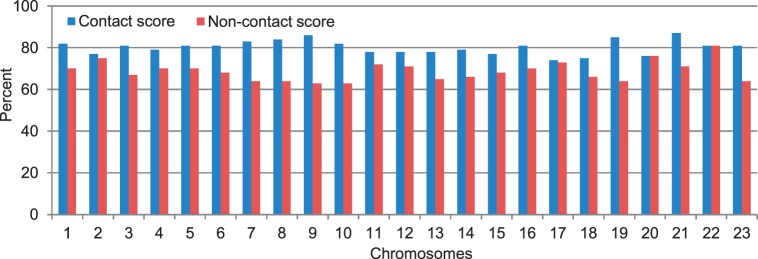
The scores of chromosomal structures at 200 KB resolution for chromosomes 1–23.

In addition to 1 MB base pair resolution, we tested our method at the higher resolution—200 KB base pairs. At resolution of 200 KB, our method can construct chromosome models when the percentages of contacts are in range (19% to 65%). We used the IF cutoff value of 2.0 to remove potentially noisy contacts after normalization. The distance thresholds used in formula (2) at 200 KB resolution were derived using the same approach as previously at 1 MB resolution. However, because the percentage of contacts at 200 KB resolution is not as high as the percentage of contacts at 1 MB resolution, 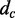 should be set at a value lower than the average distance measured at 1 MB. Furthermore, the value of 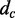 should make structures at 200 KB resolutions consistent with structures at 1 MB resolution. We tried different values and found that setting 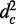 at 4.5 um satisfied this requirement. Because of the limitation of distance measurements for pairs of loci at ∼200 KB apart, 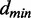 and 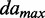 are subjectively set at some values lower than the corresponding values at 1 MB resolution. Specifically, 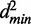 is set to 0.02 um, 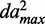 to 1.0 um and 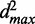 to 20 um that is the same as that at 1 MB resolution. Given this setting, we adjusted 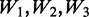 and 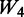 to get higher contact and non-contact scores, with more emphasis on contact scores, as the percentage of contacts at this resolution is low. Supplementary Table S4 reports the values of these four parameters for all chromosomes. Figure 9 shows contact and non-contact scores for all chromosomes.

**Figure 10.**
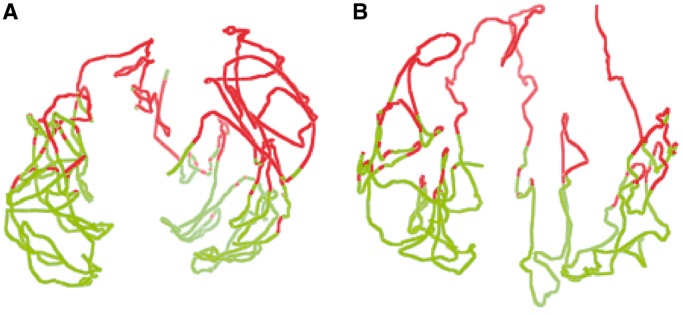
Two compartments in the models of chromosomes 1 (**A**) and 11 (**B**) at resolution of 200 KB. Red denotes the euchromatin compartment and green the heterochromatin compartment.

We also evaluated the models with the two-compartment feature as we did for the models at 1 MB. We observed that the two compartments of all chromosomal models are separable. Figure 10 illustrates the two separated compartments of the models for chromosomes 1 and 11. Supplementary Figure S4 illustrates the two compartments of the models at 200 KB for all 23 pairs of chromosomes.

**Figure 11.**
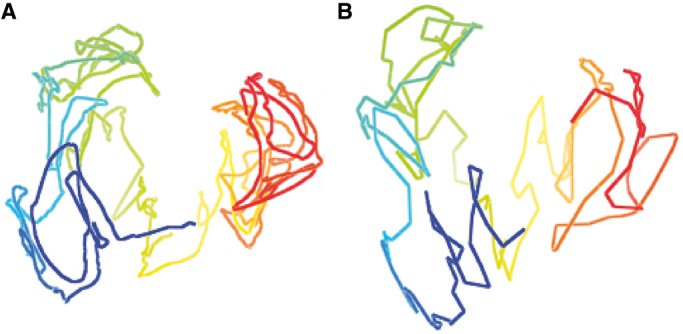
The models of chromosome 1 at resolution of 200 K (**A**) and at resolution of 1 MB (**B**).

**Figure 12.**
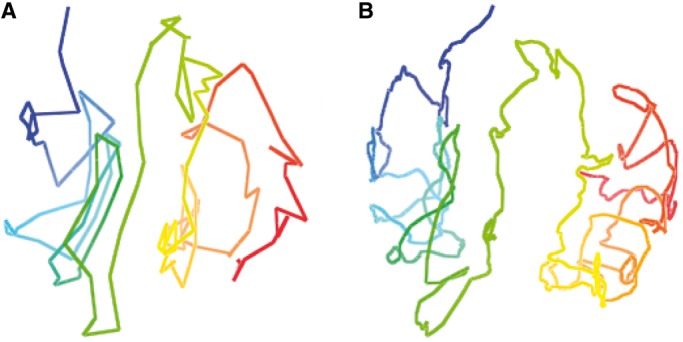
The model of chromosome 11 at resolution of 1 MB (**A**) and at resolution of 200 KB (**B**).

Finally, we compared the models of the same chromosome constructed at two different resolutions (1 MB and 200 KB). The comparison showed that models for the same chromosome at the two resolutions are consistent. Given a model at resolution of 200 KB, there is always a model in the corresponding ensemble at resolution of 1 MB sharing the same shape, demonstrating that our method is robust against the change of resolutions. Figures 11 and 12 illustrate the similarity of models of chromosomes 1 and 11 at the two resolutions. Supplementary Figure S3 compares the models at 200 KB and 1 MB side by side for all chromosomes. We found that by adjusting parameters 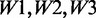 and 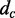, our method could always generate models at higher resolution (e.g. 200 KB) when the percentage of contacts is usually low, which are consistent with models at lower resolutions (e.g. 1 MB). This is interesting because we could use models at low resolutions to guide the construction of models at higher resolutions.

### Comparison of models constructed from the Hi-C data of the normal B-cell and the leukemia B-cell

We also tested our method on the Hi-C data of the leukemia B-cell obtained from (10). Because of the lack of FISH measurements for the leukemia B-cell, we had to use the same distance thresholds as those of the normal B-cell data, assuming that distance thresholds for the leukemia B-cell should not be different from those of the normal B-cell and that our method could tolerate some deviations in these distance thresholds. We used the same method used on the normal B-cell data to determine the IF cutoff value to remove potentially noisy contacts in the leukemia B-cell data. In the leukemia B-cell data, small chromosomes still have more interactions with each other than large chromosomes, but there are some unusual dense contacts between large chromosomes or between a small chromosome and a large chromosome, which might be due to chromosomal translocations (10). Thus, we used the IF cutoff value of 0.5, which is smaller than that for the normal B-cell, but appears to reflect interactions between chromosomes in the leukemia B-cell data well.

**Figure 13.**
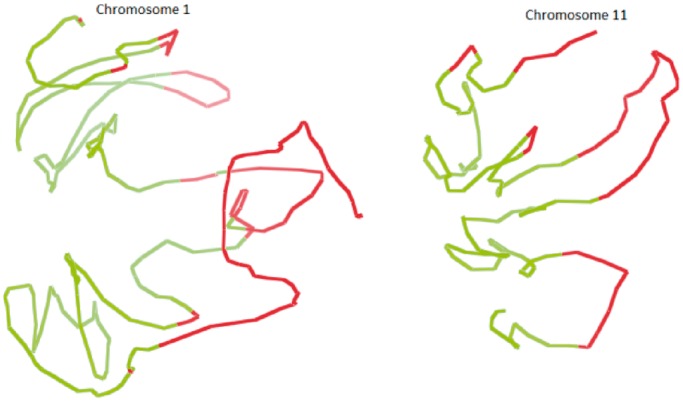
The illustration of two compartments in the models of chromosomes 1 and 11 of the leukemia B-cell.

After generating models, we evaluated them using the two-compartment feature. Figure 13 shows the two compartments in different colors of the models of chromosomes 1 and 11. Supplementary Figure S5 visualizes the two compartments of all chromosomal models in the leukemia B-cell. We observed that in the leukemia B-cell, the regions in the same compartment were also spatially clustered together and the two compartments occupied mostly separate spaces, although they might not be completely separable.

**Figure 14.**
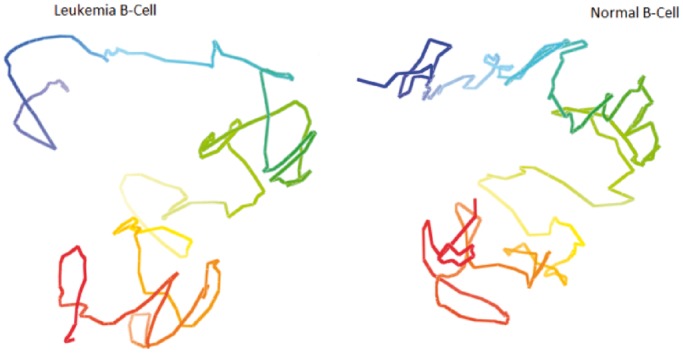
The models of chromosome 1 in the leukemia B-cell and the normal B-cell. The global topology of the two models is similar; even the models were constructed from two Hi-C data sets of two different B-cells collected by two research groups independently.

**Figure 15.**
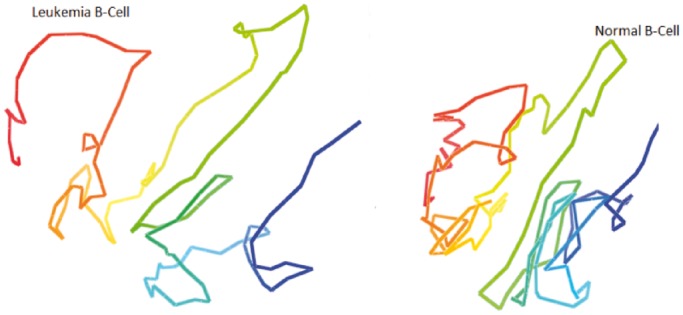
The models of chromosome 11 in the leukaemia B-cell and the normal B-cell. The global topology of the two models is similar; even the models were constructed from two Hi-C data sets of two different B-cells collected by two research groups independently.

Next, we compared the models of the normal B-cell with those of the leukemia B-cell. Figures 14 and 15 show the models of chromosomes 1 and 11 in the leukemia B-cell and the normal B-cell, respectively, side by side. We observed that the global topologies of the models of all chromosomes of the two cells had substantial similarity, even though there might be significant difference at specific loci.

**Figure 16.**
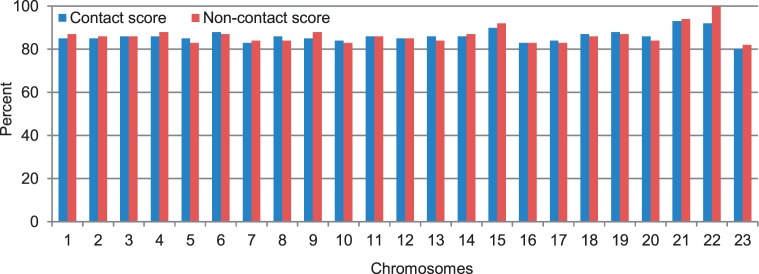
The contact scores and non-contact scores of the chromosomal models of the leukemia B-cell.

To compare the models of the two cells quantitatively, we calculated the average GDT-HA score between the models of the leukemia B-cell with the models of the normal B-cell for each chromosome. The scores range from 0.51 to 0.68 (see green bars in Figure 3). On one hand, these scores are lower than the corresponding average GDT-HA score of the models of the same chromosome of the same normal B-cell (see the blue bars in Figure 3), indicating there is some difference in the conformations of the chromosomes in the healthy and malignant B-cells; on the other hand, the scores are high enough, suggesting some similarity and conservation of chromosomal conformations in the two related human cells. Figure 16 reports contact and non-contact scores of the models for all chromosomes of the leukemia B-cell.

From the comparison of the chromosomal models of the leukemia B-cell and the normal B-cell, we also found that many local chromosomal regions that were densely packaged in the models of the normal B-cell were less densely packaged in the corresponding models of the leukemia B-cell, which might be associated with the genome-wide hypomethylation in the cancer cell (18,19). Furthermore, for both the normal and leukemia B-cells, there were many densely packaged regions in large chromosomes, but just a few in small chromosomes, which is consistent with the fact that regions in small chromosomes have more interactions with each other than in large chromosomes.

## DISCUSSION

We presented an optimization method to construct preferred 3D structural models for human chromosomes by directly satisfying the chromosomal contacts obtained from the Hi-C data sets, which is different from other distance-based chromosome model construction methods. The gradient descent optimization process was guided by a novel parameterized objective function. The experiments demonstrate that the models constructed by the method satisfy chromosomal contacts well. The chromosomal models were further validated by the two-compartment feature of human chromosomes. The experiments also showed that the method could recover unseen contacts with higher IF and was also robust against some parameter values and the incompleteness of contact information.

Moreover, our method can be used to effectively generate structures at different resolutions as long as there is the sufficient number of contacts. The models constructed at resolution of 200 KB and at resolution of 1 MB were consistent, suggesting the effectiveness of the method. Furthermore, we compared the models constructed from both the healthy and cancerous B-cells, which illustrated both the possible variation and conservation in the conformations of chromosomes in the two cells. We believe, as one of the first predicted 3D structures of human chromosomes, our models are valuable for studying human chromosome organization and its effect on cell development, cell differentiation and gene regulation. In the future, we hope to generate some FISH measurements of chromosomal distances to further validate the chromosomal models constructed by our method and calibrate the parameters in the objective function. We also plan to construct the 3D model of the whole human genome by progressively docking chromosomal models together.

## SUPPLEMENTARY DATA

Supplementary Data are available at NAR online.

## FUNDING

The National Science Foundation [grant DBI1149224 to J.C.]. Funding for open access charge: the National Science Foundation, USA.

*Conflict of interest statement*. None declared.

## Supplementary Material

Supplementary Data
