# Supplementary material for: Large-scale reconstruction of 3D structures of human chromosomes from chromosomal contact data
Source: Nucleic Acids Res. 2014 Jan 24;42(7):e52. doi: 10.1093/nar/gkt1411 (PMC3985632; doi:10.1093/nar/gkt1411)
Supplement: Supplementary Data [file supp_42_7_e52__index.html]

Large-scale reconstruction of 3D structures of human chromosomes from chromosomal contact data — Large-scale reconstruction of 3D structures of human chromosomes from chromosomal contact data — Supplementary Data 

# Large-scale reconstruction of 3D structures of human chromosomes from chromosomal contact data

## Supplementary Data

files

**Files in this Data Supplement:**

- Supplementary Data - docx file
