# Supplementary material for: Large-scale reconstruction of 3D structures of human chromosomes from chromosomal contact data
Source: Nucleic Acids Res. 2014 Jan 24;42(7):e52. doi: 10.1093/nar/gkt1411 (PMC3985632; doi:10.1093/nar/gkt1411)

**Supplementary Material**

**Table S1** Percentages of intrachromosomal and interchromosomal contacts between 23 pairs of chromosomes before removing noise contacts.

| Chr. | 1 | 2 | 3 | 4 | 5 | 6 | 7 | 8 | 9 | 10 | 11 | 12 | 13 | 14 | 15 | 16 | 17 | 18 | 19 | 20 | 21 | 22 | 23 |
| --- | --- | --- | --- | --- | --- | --- | --- | --- | --- | --- | --- | --- | --- | --- | --- | --- | --- | --- | --- | --- | --- | --- | --- |
| 1 | 99 | 97 | 97 | 97 | 98 | 97 | 97 | 98 | 97 | 97 | 98 | 98 | 96 | 98 | 98 | 97 | 97 | 97 | 96 | 98 | 97 | 97 | 97 |
| 2 | 97 | 99 | 97 | 97 | 98 | 97 | 97 | 97 | 97 | 97 | 98 | 97 | 96 | 97 | 98 | 97 | 97 | 97 | 95 | 97 | 98 | 97 | 97 |
| 3 | 97 | 97 | 99 | 97 | 98 | 97 | 97 | 97 | 97 | 97 | 98 | 97 | 96 | 97 | 97 | 97 | 96 | 97 | 95 | 97 | 98 | 97 | 97 |
| 4 | 97 | 97 | 97 | 99 | 98 | 97 | 97 | 97 | 97 | 97 | 98 | 97 | 96 | 97 | 97 | 96 | 96 | 97 | 93 | 97 | 97 | 95 | 97 |
| 5 | 98 | 98 | 98 | 98 | 99 | 98 | 97 | 98 | 97 | 98 | 98 | 98 | 96 | 98 | 98 | 97 | 97 | 97 | 95 | 97 | 98 | 96 | 97 |
| 6 | 97 | 97 | 97 | 97 | 98 | 99 | 97 | 97 | 97 | 97 | 98 | 97 | 96 | 97 | 98 | 97 | 96 | 97 | 94 | 97 | 97 | 96 | 97 |
| 7 | 97 | 97 | 97 | 97 | 97 | 97 | 99 | 97 | 97 | 97 | 97 | 97 | 96 | 97 | 97 | 97 | 96 | 96 | 95 | 97 | 97 | 96 | 97 |
| 8 | 98 | 97 | 97 | 97 | 98 | 97 | 97 | 99 | 97 | 98 | 98 | 98 | 96 | 97 | 98 | 97 | 97 | 97 | 95 | 97 | 98 | 97 | 97 |
| 9 | 97 | 97 | 97 | 97 | 97 | 97 | 97 | 97 | 99 | 97 | 97 | 97 | 95 | 97 | 98 | 97 | 97 | 97 | 95 | 98 | 97 | 97 | 96 |
| 10 | 97 | 97 | 97 | 97 | 98 | 97 | 97 | 98 | 97 | 99 | 98 | 98 | 96 | 98 | 98 | 97 | 98 | 97 | 97 | 98 | 98 | 98 | 97 |
| 11 | 98 | 98 | 98 | 98 | 98 | 98 | 97 | 98 | 97 | 98 | 99 | 98 | 97 | 98 | 99 | 98 | 98 | 98 | 96 | 98 | 98 | 98 | 98 |
| 12 | 98 | 97 | 97 | 97 | 98 | 97 | 97 | 98 | 97 | 98 | 98 | 99 | 96 | 98 | 98 | 97 | 97 | 97 | 96 | 98 | 98 | 97 | 97 |
| 13 | 96 | 96 | 96 | 96 | 96 | 96 | 96 | 96 | 95 | 96 | 97 | 96 | 99 | 96 | 97 | 96 | 95 | 97 | 92 | 96 | 97 | 94 | 96 |
| 14 | 98 | 97 | 97 | 97 | 98 | 97 | 97 | 97 | 97 | 98 | 98 | 98 | 96 | 99 | 98 | 98 | 97 | 98 | 96 | 98 | 98 | 97 | 97 |
| 15 | 98 | 98 | 97 | 97 | 98 | 98 | 97 | 98 | 98 | 98 | 99 | 98 | 97 | 98 | 99 | 98 | 98 | 98 | 98 | 98 | 98 | 99 | 97 |
| 16 | 97 | 97 | 97 | 96 | 97 | 97 | 97 | 97 | 97 | 97 | 98 | 97 | 96 | 98 | 98 | 99 | 98 | 98 | 98 | 98 | 98 | 98 | 97 |
| 17 | 97 | 97 | 96 | 96 | 97 | 96 | 96 | 97 | 97 | 98 | 98 | 97 | 95 | 97 | 98 | 98 | 99 | 97 | 99 | 98 | 97 | 99 | 96 |
| 18 | 97 | 97 | 97 | 97 | 97 | 97 | 96 | 97 | 97 | 97 | 98 | 97 | 97 | 98 | 98 | 98 | 97 | 99 | 96 | 97 | 98 | 97 | 97 |
| 19 | 96 | 95 | 95 | 93 | 95 | 94 | 95 | 95 | 95 | 97 | 96 | 96 | 92 | 96 | 98 | 98 | 99 | 96 | 99 | 98 | 97 | 99 | 94 |
| 20 | 98 | 97 | 97 | 97 | 97 | 97 | 97 | 97 | 98 | 98 | 98 | 98 | 96 | 98 | 98 | 98 | 98 | 97 | 98 | 99 | 99 | 99 | 97 |
| 21 | 97 | 98 | 98 | 97 | 98 | 97 | 97 | 98 | 97 | 98 | 98 | 98 | 97 | 98 | 98 | 98 | 97 | 98 | 97 | 99 | 100 | 98 | 97 |
| 22 | 97 | 97 | 97 | 95 | 96 | 96 | 96 | 97 | 97 | 98 | 98 | 97 | 94 | 97 | 99 | 98 | 99 | 97 | 99 | 99 | 98 | 100 | 96 |
| 23 | 97 | 97 | 97 | 97 | 97 | 97 | 97 | 97 | 96 | 97 | 98 | 97 | 96 | 97 | 97 | 97 | 96 | 97 | 94 | 97 | 97 | 96 | 99 |

**Table S2** Percentages of intrachromosomal and interchromosomal contacts between 23 pairs of chromosomes after removing noise contacts.

| Chr. | 1 | 2 | 3 | 4 | 5 | 6 | 7 | 8 | 9 | 10 | 11 | 12 | 13 | 14 | 15 | 16 | 17 | 18 | 19 | 20 | 21 | 22 | 23 |
| --- | --- | --- | --- | --- | --- | --- | --- | --- | --- | --- | --- | --- | --- | --- | --- | --- | --- | --- | --- | --- | --- | --- | --- |
| 1 | 49 | 0 | 0 | 1 | 0 | 0 | 0 | 0 | 1 | 0 | 0 | 0 | 1 | 1 | 0 | 1 | 1 | 0 | 1 | 0 | 1 | 1 | 0 |
| 2 | 0 | 55 | 0 | 0 | 0 | 0 | 0 | 0 | 1 | 0 | 0 | 0 | 1 | 0 | 0 | 0 | 0 | 0 | 0 | 0 | 1 | 0 | 0 |
| 3 | 0 | 0 | 66 | 1 | 0 | 1 | 1 | 1 | 1 | 0 | 1 | 1 | 1 | 1 | 0 | 0 | 0 | 0 | 0 | 0 | 1 | 0 | 1 |
| 4 | 1 | 0 | 1 | 65 | 1 | 1 | 1 | 1 | 1 | 1 | 1 | 1 | 2 | 1 | 0 | 0 | 0 | 1 | 1 | 1 | 2 | 1 | 1 |
| 5 | 0 | 0 | 0 | 1 | 64 | 1 | 0 | 1 | 1 | 0 | 1 | 1 | 1 | 1 | 0 | 0 | 0 | 1 | 1 | 0 | 2 | 0 | 1 |
| 6 | 0 | 0 | 1 | 1 | 1 | 70 | 1 | 1 | 1 | 0 | 1 | 1 | 1 | 1 | 0 | 0 | 1 | 0 | 1 | 0 | 1 | 0 | 1 |
| 7 | 0 | 0 | 1 | 1 | 0 | 1 | 74 | 1 | 1 | 0 | 1 | 1 | 1 | 1 | 0 | 1 | 1 | 1 | 1 | 1 | 1 | 1 | 1 |
| 8 | 0 | 0 | 1 | 1 | 1 | 1 | 1 | 77 | 1 | 0 | 1 | 1 | 1 | 1 | 0 | 1 | 1 | 1 | 1 | 0 | 1 | 0 | 1 |
| 9 | 1 | 1 | 1 | 1 | 1 | 1 | 1 | 1 | 75 | 1 | 1 | 1 | 1 | 2 | 1 | 2 | 3 | 2 | 3 | 2 | 3 | 3 | 1 |
| 10 | 0 | 0 | 0 | 1 | 0 | 0 | 0 | 0 | 1 | 78 | 0 | 0 | 1 | 0 | 1 | 0 | 0 | 1 | 0 | 1 | 2 | 1 | 0 |
| 11 | 0 | 0 | 1 | 1 | 1 | 1 | 1 | 1 | 1 | 0 | 77 | 1 | 1 | 1 | 0 | 1 | 2 | 1 | 2 | 1 | 2 | 2 | 1 |
| 12 | 0 | 0 | 1 | 1 | 1 | 1 | 1 | 1 | 1 | 0 | 1 | 75 | 1 | 1 | 0 | 1 | 1 | 1 | 1 | 1 | 2 | 1 | 1 |
| 13 | 1 | 1 | 1 | 2 | 1 | 1 | 1 | 1 | 1 | 1 | 1 | 1 | 94 | 2 | 1 | 1 | 1 | 2 | 1 | 1 | 6 | 1 | 1 |
| 14 | 1 | 0 | 1 | 1 | 1 | 1 | 1 | 1 | 2 | 0 | 1 | 1 | 2 | 86 | 2 | 2 | 2 | 2 | 2 | 2 | 5 | 4 | 1 |
| 15 | 0 | 0 | 0 | 0 | 0 | 0 | 0 | 0 | 1 | 1 | 0 | 0 | 1 | 2 | 89 | 2 | 2 | 1 | 2 | 2 | 4 | 3 | 0 |
| 16 | 1 | 0 | 0 | 0 | 0 | 0 | 1 | 1 | 2 | 0 | 1 | 1 | 1 | 2 | 2 | 86 | 4 | 2 | 7 | 3 | 6 | 7 | 0 |
| 17 | 1 | 0 | 0 | 0 | 0 | 1 | 1 | 1 | 3 | 0 | 2 | 1 | 1 | 2 | 2 | 4 | 85 | 1 | 11 | 4 | 6 | 10 | 0 |
| 18 | 0 | 0 | 0 | 1 | 1 | 0 | 1 | 1 | 2 | 1 | 1 | 1 | 2 | 2 | 1 | 2 | 1 | 97 | 1 | 2 | 8 | 2 | 1 |
| 19 | 1 | 0 | 0 | 1 | 1 | 1 | 1 | 1 | 3 | 0 | 2 | 1 | 1 | 2 | 2 | 7 | 11 | 1 | 92 | 4 | 10 | 18 | 0 |
| 20 | 0 | 0 | 0 | 1 | 0 | 0 | 1 | 0 | 2 | 1 | 1 | 1 | 1 | 2 | 2 | 3 | 4 | 2 | 4 | 93 | 8 | 11 | 0 |
| 21 | 1 | 1 | 1 | 2 | 2 | 1 | 1 | 1 | 3 | 2 | 2 | 2 | 6 | 5 | 4 | 6 | 6 | 8 | 10 | 8 | 94 | 16 | 1 |
| 22 | 1 | 0 | 0 | 1 | 0 | 0 | 1 | 0 | 3 | 1 | 2 | 1 | 1 | 4 | 3 | 7 | 10 | 2 | 18 | 11 | 16 | 96 | 0 |
| 23 | 0 | 0 | 1 | 1 | 1 | 1 | 1 | 1 | 1 | 0 | 1 | 1 | 1 | 1 | 0 | 0 | 0 | 1 | 0 | 0 | 1 | 0 | 83 |

**Table S3** The weight parameters and the percent of contacts after removing nose for 23 pairs of chromosomes at 1MB resolution.

| Chromosome | Percentage of contact pairs | $W_{1}$ | $W_{2}$ | $W_{3}$ | $W_{4}$ |
| --- | --- | --- | --- | --- | --- |
| 1 | 49% | 2.0 | 1.0 | 1.0 | 1.0 |
| 2 | 55% | 2.0 | 1.0 | 1.0 | 1.0 |
| 3 | 66% | 1.2 | 1.0 | 1.0 | 1.0 |
| 4 | 65% | 1.0 | 1.0 | 1.0 | 1.0 |
| 5 | 64% | 1.0 | 1.1 | 1.1 | 1.1 |
| 6 | 70% | 1.0 | 1.2 | 1.2 | 1.2 |
| 7 | 74% | 1.0 | 1.5 | 1.5 | 1.5 |
| 8 | 77% | 1.0 | 1.6 | 1.6 | 1.6 |
| 9 | 75% | 1.0 | 2.2 | 2.2 | 2.2 |
| 10 | 78% | 1.0 | 1.8 | 1.8 | 1.8 |
| 11 | 77% | 1.0 | 1.8 | 1.8 | 1.8 |
| 12 | 75% | 1.0 | 1.5 | 1.5 | 1.5 |
| 13 | 94% | 1.0 | 4.6 | 4.6 | 4.6 |
| 14 | 86% | 1.0 | 3.5 | 3.5 | 3.5 |
| 15 | 89% | 1.0 | 3.0 | 3.0 | 3.0 |
| 16 | 86% | 1.0 | 3.9 | 3.9 | 3.9 |
| 17 | 85% | 1.0 | 3.0 | 3.0 | 3.0 |
| 18 | 97% | 1.0 | 9.5 | 9.5 | 9.5 |
| 19 | 92% | 1.0 | 4.0 | 4.0 | 4.0 |
| 20 | 93% | 1.0 | 5.0 | 5.0 | 5.0 |
| 21 | 94% | 1.0 | 6.0 | 6.0 | 6.0 |
| 22 | 96% | 1.0 | 6.0 | 6.0 | 6.0 |
| 23 | 83% | 1.0 | 2.0 | 2.0 | 2.0 |

The weight parameters are used with the objective function below to guide the reconstruction of chromosomal structures:

$Fn=\sum_{\begin{aligned} contacts \\ \left( i,j \right), &\left| i-j \right|\neq1&& \end{aligned}} \left( W_{1}* \tanh\left( d_{c}^{2}-d_{ij}^{2} \right)*N_{ij}+W_{2} *\frac{\tanh\left( d_{ij}^{2}-d_{min}^{2} \right)}{totalIF} \right)+\sum_{\begin{aligned} non-contacts \\ \left( i,j \right), \left| i-j \right|\neq1 \end{aligned}} \left( W_{3} *\frac{\tanh\left( d_{max}^{2}-d_{ij}^{2} \right)}{totalIF}+W_{4} *\frac{\tanh\left( d_{ij}^{2}-d_{c}^{2} \right)}{totalIF} \right) +\sum_{\left| i-j \right|=1} \left( W_{1}* \frac{IF_{max}}{totalIF} *\frac{\tanh\left( {da}_{max}^{2}-d_{ij}^{2} \right)}{1}+W_{2} *\frac{\tanh\left( d_{ij}^{2}-d_{min}^{2} \right)}{totalIF} \right)$

The equation is designed artificially in order to satisfy contacts of higher probability with higher priority, while maintaining the distance restraints between two adjacent units in a polymer. The first term enforces the minimum distance and the predetermined distance threshold between contacted regions; the second term enforces the predetermined distance threshold and the maximum distance between non contacted regions; and the third term enforces the minimum and maximum distance restraints between two adjacent regions.

Figure S1 The plots of IFs of missing contacts and recovered contacts. Contacts were sorted according to Ifs. The plots show that contacts with higher IF most likely were recovered.


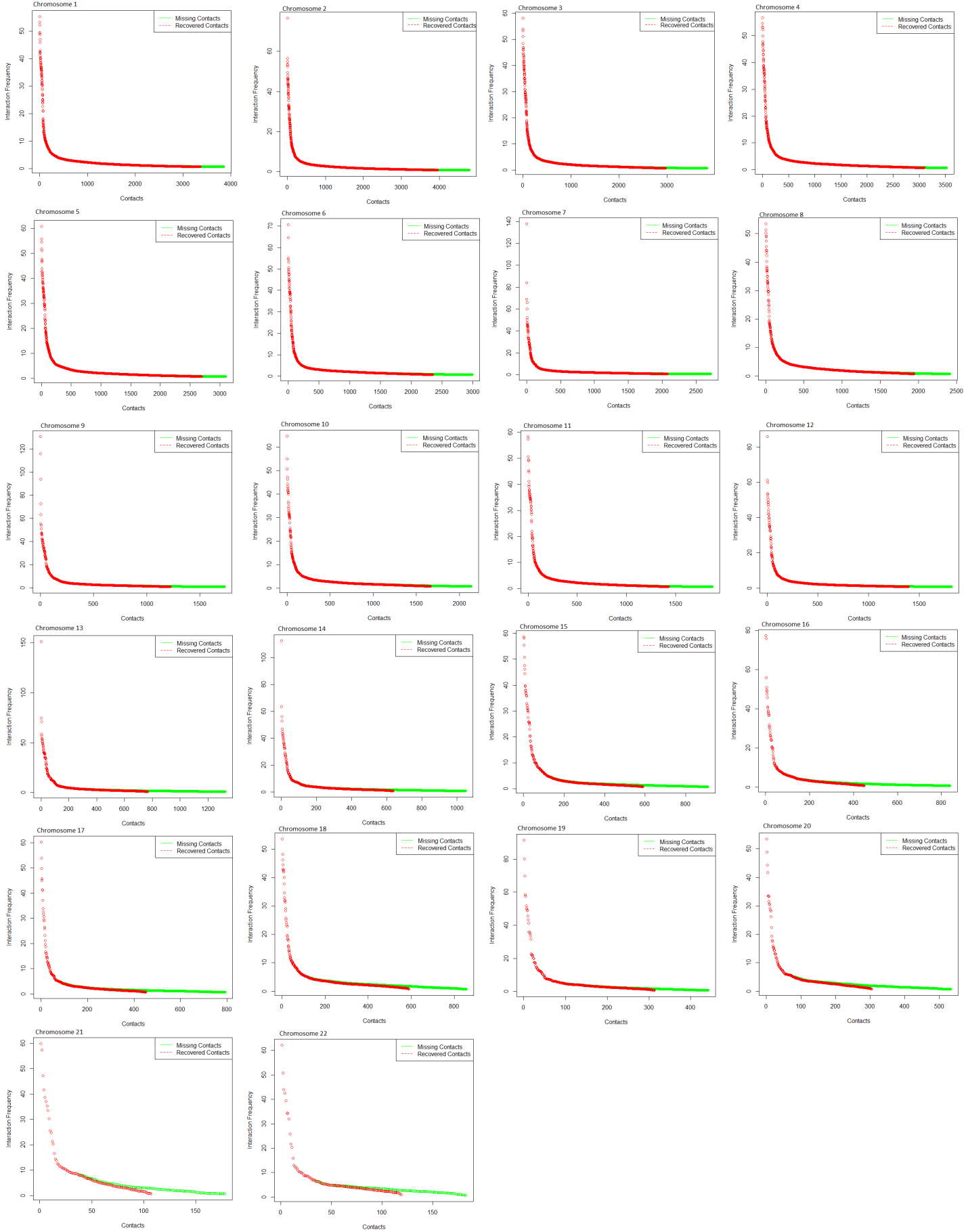


Figure S2 Two compartments in different colors of all chromosome models at 1MB resolution of the normal B-cell.Red compartments are more closely associated with open, accessible, actively transcribed chromatins (euchromatin compartment).


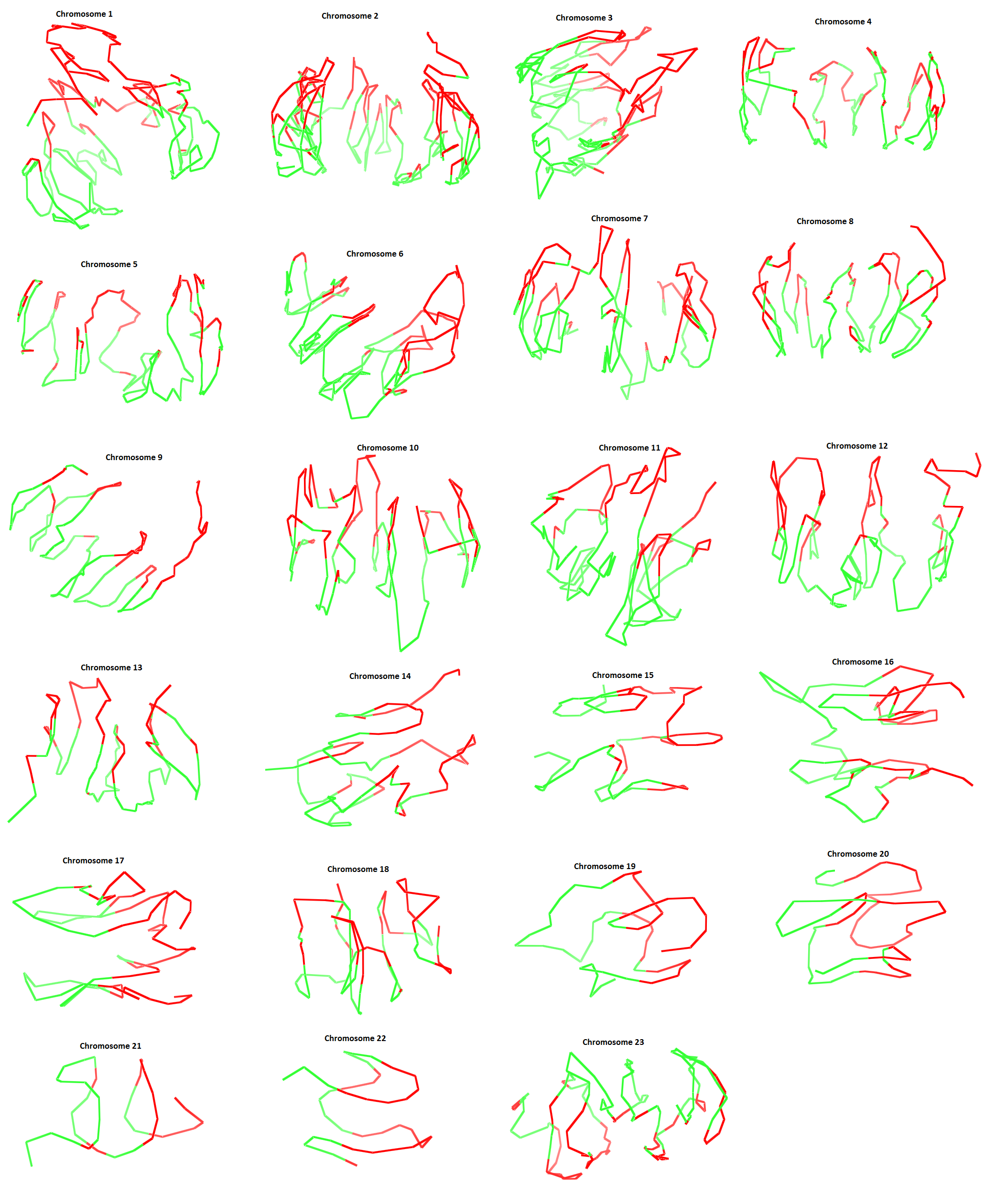


Table S4 – Percentage of contact pairs and weight parameters used to generate structures at resolution of 200KB

| Chr. | Percentage of contact pairs (%) | $W_{1}$ | $W_{2}$ | $W_{3}$ | $W_{4}$ |
| --- | --- | --- | --- | --- | --- |
| 1 | 19 | 1.0 | 0.4 | 0.4 | 0.4 |
| 2 | 21 | 1.0 | 0.7 | 0.7 | 0.7 |
| 3 | 24 | 1.0 | 0.65 | 0.65 | 0.65 |
| 4 | 28 | 1.0 | 1.0 | 1.0 | 1.0 |
| 5 | 27 | 1.0 | 0.8 | 0.8 | 0.8 |
| 6 | 28 | 1.0 | 0.8 | 0.8 | 0.8 |
| 7 | 30 | 1.0 | 0.8 | 0.8 | 0.8 |
| 8 | 33 | 1.0 | 0.85 | 0.85 | 0.85 |
| 9 | 33 | 1.0 | 0.9 | 0.9 | 0.9 |
| 10 | 32 | 1.0 | 1.1 | 1.1 | 1.1 |
| 11 | 30 | 1.0 | 1.0 | 1.0 | 1.0 |
| 12 | 30 | 1.0 | 1.0 | 1.0 | 1.0 |
| 13 | 46 | 1.0 | 2.1 | 2.1 | 2.1 |
| 14 | 40 | 1.0 | 1.2 | 1.2 | 1.2 |
| 15 | 40 | 1.0 | 1.5 | 1.5 | 1.5 |
| 16 | 43 | 1.0 | 1.6 | 1.6 | 1.6 |
| 17 | 37 | 1.0 | 1.2 | 1.2 | 1.2 |
| 18 | 52 | 1.0 | 3.0 | 3.0 | 3.0 |
| 19 | 53 | 1.0 | 2.8 | 2.8 | 2.8 |
| 20 | 51 | 1.0 | 2.6 | 2.6 | 2.6 |
| 21 | 65 | 1.0 | 3.8 | 3.8 | 3.8 |
| 22 | 65 | 1.0 | 5.2 | 5.2 | 5.2 |
| 23 | 38 | 1.0 | 1.0 | 1.0 | 1.0 |

Figure S3 Comparison of structures at 1MB resolution (on the left) and 200KB resolution (on the right).


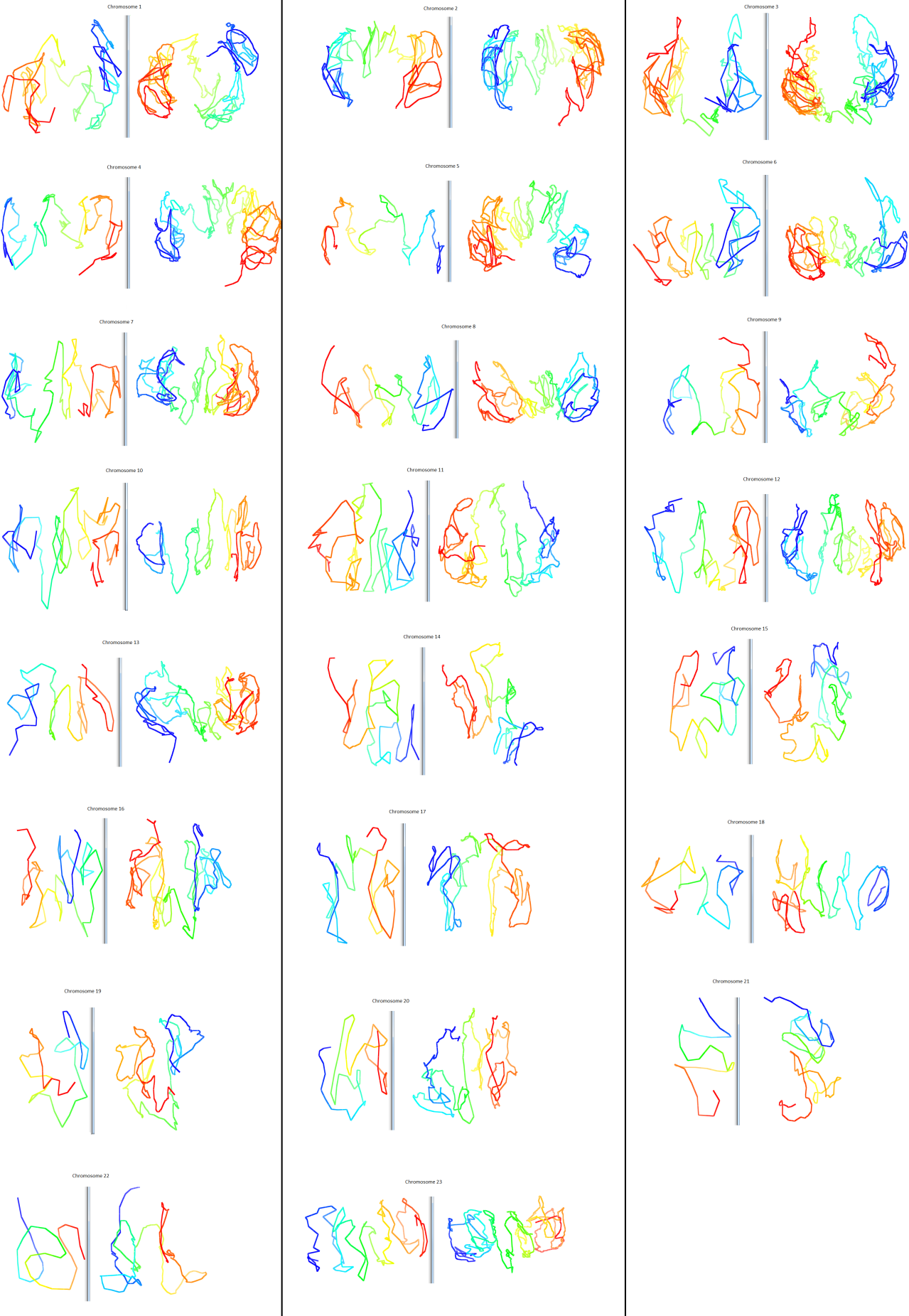


Figure S4 Two compartments in different colors in chromosomal models at 200KB resolution of the normal B-cell. The color here is arbitrary for each chromosome.


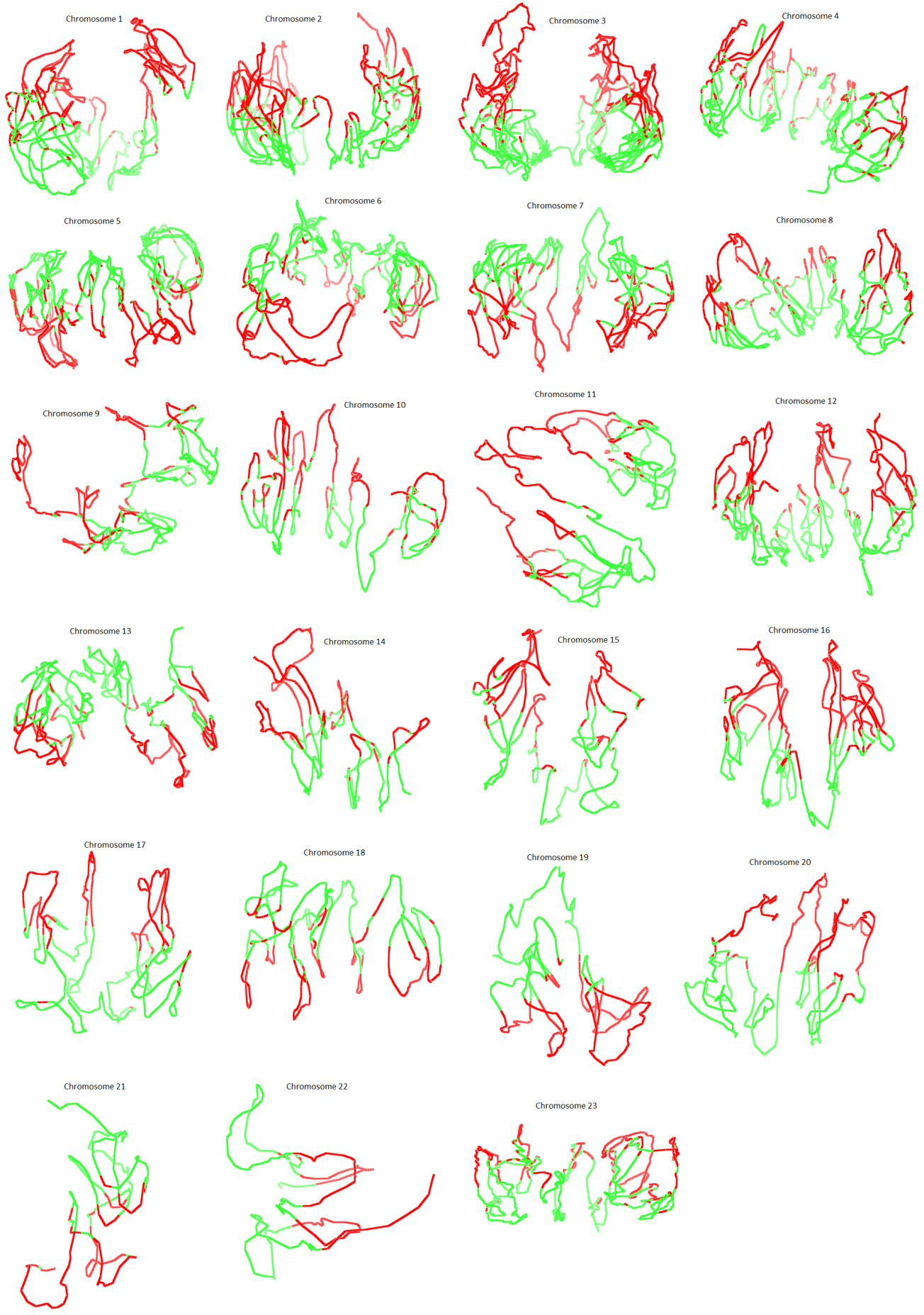


Figure S5 Two compartments in different colors in chromosomal models of the leukemia B-cell at 1MB resolution. The color here is arbitrary. T the
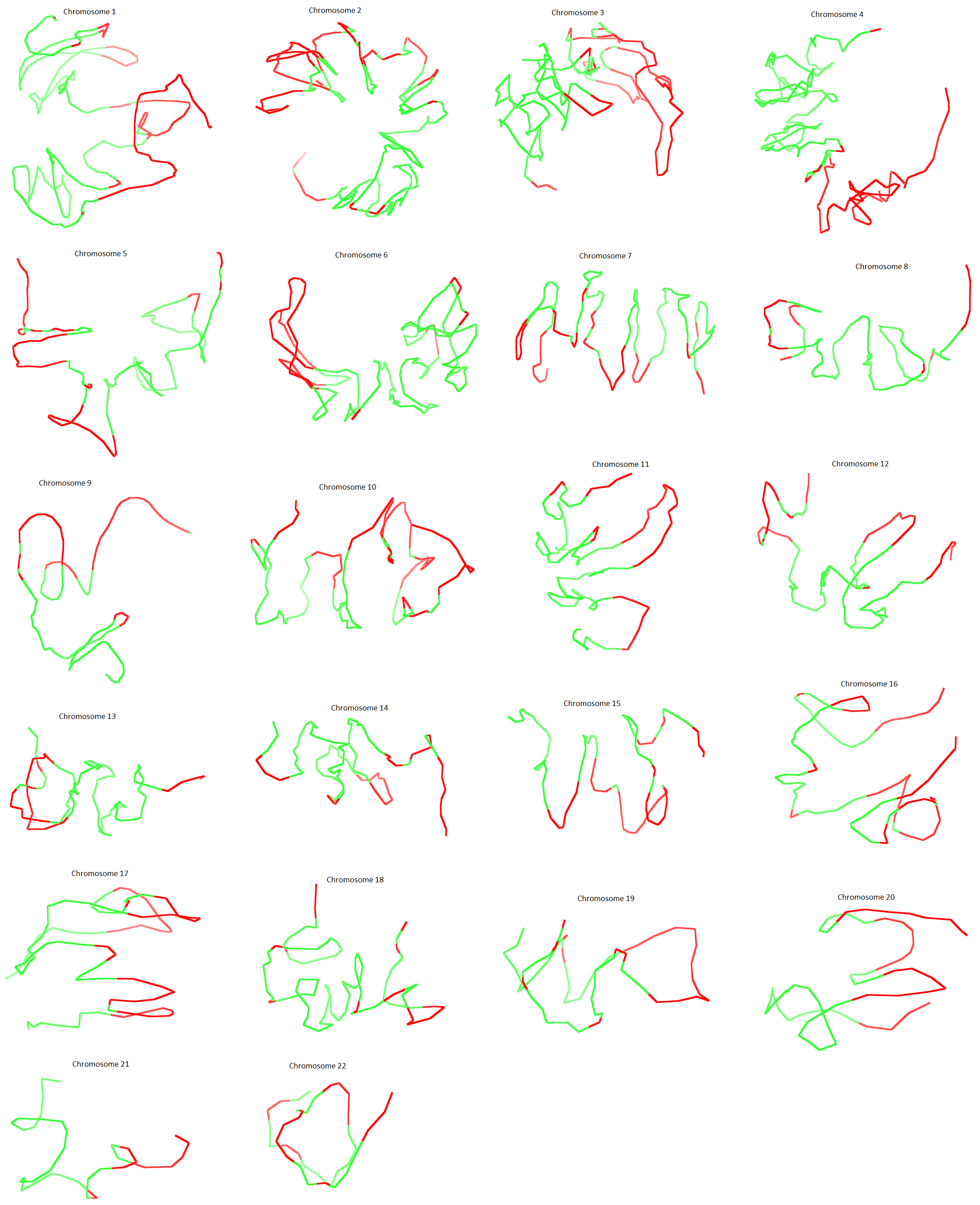


Figure S6 Comparison of the models of the leukemia B-cell and the normal B-cell


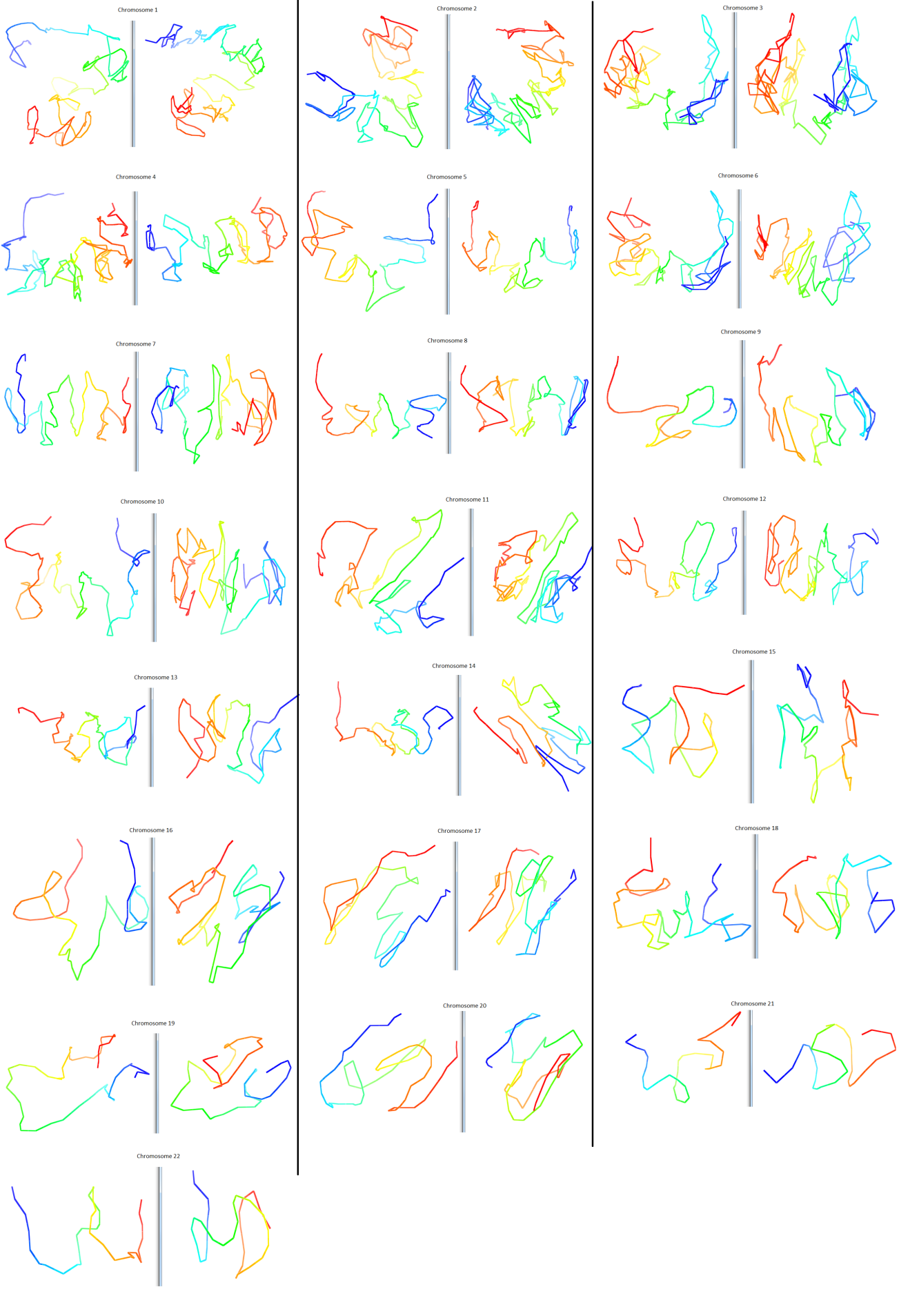

Supplement: Supplementary Data [file supp_gkt1411_nar-03108-met-g-2013-File002.docx]
